# Altered maturation of peripheral blood dendritic cells in patients with breast cancer

**DOI:** 10.1038/sj.bjc.6601243

**Published:** 2003-10-14

**Authors:** S Della Bella, M Gennaro, M Vaccari, C Ferraris, S Nicola, A Riva, M Clerici, M Greco, M L Villa

**Affiliations:** 1 Dipartimento di Scienze e Tecnologie Biomediche, Cattedra di Immunologia, Università degli Studi di Milano, LITA Segrate, via F.lli Cervi 93, Segrate (MI) 20090, Italy; 2 Unità Operativa di Senologia, Istituto Nazionale per lo Studio e la Cura dei Tumori, via Venezian 1, Milano 20133, Italy; 3 Dipartimento di Scienze Precliniche, Cattedra di Immunologia, Università degli Studi di Milano, LITA Vialba, Ospedale Sacco, via GB Grassi 74, Milano 20156, Italy

**Keywords:** tumour immunity, dendritic cells, cell surface molecules, cytokines

## Abstract

Tumours have at least two mechanisms that can alter dendritic cell (DC) maturation and function. The first affects the ability of haematopoietic progenitors to differentiate into functional DCs; the second affects their differentiation from CD14+ monocytes, promoting an early but dysfunctional maturation. The aim of this study was to evaluate the *in vivo* relevance of these pathways in breast cancer patients. For this purpose, 53 patients with invasive breast cancer were compared to 68 healthy controls. To avoid isolation or culture procedures for enrichment of DCs, analyses were directly performed by flow cytometry on whole-blood samples. The expression of surface antigens and intracellular accumulation of regulatory cytokines upon LPS stimulation were evaluated. The number of DCs, and in particular of the myeloid subpopulation, was markedly reduced in cancer patients (*P*<0.001). Patient DCs were characterized by a more mature phenotype compared with controls (*P*=0.016), and had impaired production of IL-12 (*P*<0.001). These alterations were reverted by surgical resection of the tumour. To investigate the possible role of some tumour-related immunoactive soluble factors, we measured the plasmatic levels of vascular endothelial growth factor, IL-10 and spermine. A significant inverse correlation between spermine concentration and the percentage of DCs expressing IL-12 was found. Evidence was also obtained that *in vitro* exposure of monocyte-derived DCs to spermine promoted their activation and maturation, and impaired their function. Taken together, our results suggest that both the above-described mechanisms could concomitantly act in breast cancer to affect DC differentiation, and that spermine could be a mediator of dysfunctional maturation of DCs.

Dentritic cells (DCs) represent the most potent professional antigen-presenting cells, because of their widespread localisation in all sites of antigen entry, their ability to process tumour antigens and present them on HLA class II as well as class I molecules, their high expression of costimulatory molecules, and their production of regulatory cytokines (Rescigno *et al*, 1999; Larsson *et al*, 2001). Owing to the unique ability of DCs to stimulate naive T lymphocytes and therefore to initiate tumour-specific cytotoxic immune responses (Croft *et al*, 1992), these cells are critical elements of antitumour immunity induction.

Tumours have at least two mechanisms by which they can alter DC maturation and function. The first one affects the ability of haematopoietic progenitor cells to differentiate into functional DCs during the early stages of their maturation. *In vitro*, it has been demonstrated that factors that can be produced by tumour cells, including vascular endothelial growth factor (VEGF), M-CSF and IL-6, inhibit DC maturation from CD34+ cells (Gabrilovich *et al*, 1996; Menetrier-Caux *et al*, 1998). *In vivo*, tumour-derived VEGF has been proposed as the most likely factor to affect the early stage of DC maturation in the bone marrow (Ishida *et al*, 1998; Gabrilovich *et al*, 1999). In breast, head and neck and lung cancer, the inhibitory effects of VEGF on DC differentiation have been described to cause a decrease of PB DCs closely associated with accumulation of immature myeloid cells lacking markers of mature haematopoietic cells (Almand *et al*, 2000). The second mechanism by which tumours can alter DC maturation affects their differentiation from CD14+ monocyte precursors. This pathway, that is also mediated by soluble tumour-derived factors, acts by promoting an early but dysfunctional maturation of DCs. In particular, it has been demonstrated *in vitro* that the addition of tumour supernatants during DC differentiation from PB monocytes induces the generation of DCs that are more mature and that display a diminished capacity to take up antigens and to produce regulatory cytokines, and do not develop full allostimulatory activity (Kiertscher *et al*, 2000).

Whereas several tumour-derived soluble factors are known to affect DC differentiation from CD34+ cells (Gabrilovich *et al*, 1996; Menetrier-Caux *et al*, 1998), the mediators leading to the premature phenotypic maturation of monocyte-derived DCs still remain to be determined. A number of tumour-derived factors with the capacity of altering the maturation of CD34+or CD14+precursors, including VEGF, TGF-*β*, IL-10 and PGE_2_, have been excluded to be implicated in this pathway (Kiertscher *et al*, 2000). Polyamines are potential candidates, because they are important modulators of immune functions and occur at high concentrations in actively proliferating tissues (Seiler and Atanassov, 1994; Zhang *et al*, 1997). Spermine, in particular, has been suggested to contribute to the decreased immune reaction against tumours. In fact, elevated levels of spermine have been reported in the plasma and urine of patients with tumours, including breast cancer (Chaisiri *et al*, 1979; Lee *et al*, 1998). Furthermore, spermine has recently been demonstrated to inhibit the production of IL-12 by *in vitro*-stimulated macrophages (Haskò *et al*, 2000).

The aim of the present study was to evaluate the *in vivo* relevance of the above-described pathways followed by tumours to alter DC maturation and function. For this purpose, we enumerated and functionally characterised DCs in the peripheral blood of patients with breast cancer. To avoid isolation or culture procedures for the enrichment of DCs that may induce the selection of particular cell subsets and/or phenotypic and functional modifications of DCs present in the sample, our analyses were directly performed by flow cytometry on whole-blood samples. Our results confirmed that the number of DCs is decreased in the peripheral blood of cancer patients. In particular, the myeloid CD11c+ subpopulation of DCs was dramatically reduced. Furthermore, DCs from cancer patients were characterised by a more mature immunophenotype compared with controls, and had impaired production of IL-12. Therefore, they appeared affected by a dysfunctional maturation similar to that induced *in vitro* by tumour supernatants during DC maturation from monocyte precursors (Kiertscher *et al*, 2000). These alterations in the maturation of DCs were completely reverted by surgical resection of the tumour. *In vitro* evidence provided in this study suggests that spermine could be a possible mediator by which breast cancer affects DC functions.

## MATERIALS AND METHODS

### Patient selection

In all, 53 patients aged 29–86 (mean 58) years with histologically confirmed invasive breast cancer were enrolled into the study. Peripheral venous heparinised blood samples were obtained from all patients before any surgical, chemotherapic and/or radiant treatment. Staging was performed in accordance with the American Joint Committee on Cancer criteria (Sobin and Witterkind, 2002). A total of 15 patients had stage I disease, 16 had stage IIA and 22 had stage IIB. In all 68, sex- and age-matched healthy donors (range 24–88, mean 55 years) were used as controls. Six age-matched patients with benign tumour of the breast and six with *in situ* breast carcinoma were also included. Informed consent was obtained from all individuals.

### Immunophenotypic analysis and counting of peripheral blood cells

Whole peripheral blood samples (1 × 10^6^ cells in 100 *μ*l test^−1^) were analysed by direct immunofluorescence using three-colour stainings with mAbs directly conjugated with fluorochromes FITC, PE and PerCP. Erythrocytes were lysed after staining, using FACS Lysing Solution (Becton Dickinson, San Jose, CA, USA) according to the manufacturer's instructions. DCs were identified as positive for anti-HLA-DR PerCP (Becton Dickinson) and negative for a mixture of FITC-conjugated mAbs (Caltag Laboratoires, Burlingame, CA, USA) specific for lineage markers on T cells (CD3), B cells (CD19, CD20), NK cells (CD16) and monocytes (CD14). Anti-CD11c PE (Caltag Laboratoires) or anti-IL3R*α* (CD123) PE (Pharmingen, San Diego, CA, USA) were used for identification of myeloid and plasmacytoid DC subpopulations, respectively. Cells labelled with isotype control mAbs were included to determine background fluorescence. All operations were done at 4°C. Three-colour analysis was performed using a FACScan flow cytometer (Becton Dickinson) using CellQuest software (Macintosh). Since PB DCs are characterised by forward scatter (FSc) similar to monocytes and side scatter (SSc) similar to lymphocytes (Upham *et al*, 2000), an acquisition gate was established based on FSc and SSc, which included both the lymphocyte and monocyte populations (mononuclear cells), but excluded most granulocytes and debris. Owing to the low frequency of lineage-/HLA-DR+ cells, we routinely collected 50 000 events to visualise and gate on this population more easily. Conventional haematological parameters in comparable blood samples were also measured using an ADVIA 120 automated haematology analyser (Bayer Diagnostics, Basingstoke, Berkshire, UK). Estimates of the absolute numbers of DCs in blood were calculated from the proportion of DCs recorded in the mononuclear gate determined by flow cytometry multiplied by absolute mononuclear cell count. The assumption was that lymphocytes and monocytes, as determined by the haematology cell counter, together constitute the mononuclear fraction identified by flow cytometry. PE-conjugated anti-CD80, CD83, CD86 (Pharmingen) and CD119 (Caltag Laboratoires) were used to evaluate the activation and maturation states of Lin-/HLA-DR+ DCs, directly in fresh whole-blood samples or in blood samples diluted v v^−1^ in RPMI 1640 medium (Euroclone, Wetherby, West York, UK), and incubated for 5 h with or without LPS (100 ng ml^−1^; from *Escherichia coli*, serotype 055:B5; Sigma). The frequency of DC precursors in the peripheral blood was determined by direct immunofluorescent staining with FITC-conjugated anti-CD34 or CD14 antibodies (Caltag Laboratoires).

### Cytoplasmic cytokine expression

Expression of the cytokines IL-12 and IL-10 in the cytoplasm of PB Lin-/HLA-DR+DCs was determined by flow cytometry. Whole peripheral blood (500 *μ*l) supplemented with 500 *μ*l of RPMI 1640 medium was incubated for 5 h with or without 100 ng ml^−1^ LPS. The protein transport inhibitor brefeldin A (BFA) (10 *μ*g ml^−1^; Sigma, St Louis, MO, USA) was added during the last 4 h, to allow intracellular accumulation of cytokines. Incubation was performed at 37°C in a 5% CO_2_ humid atmosphere in a sterile environment. Doses of BFA and LPS, and incubation times, were optimised in preliminary experiments. At the end of the incubation period, samples were aliquoted and labelled with appropriate combinations of mAbs for staining of surface markers. Cells were then fixed, permeabilised and stained with cytokine-directed mAbs anti-IL-12 PE and anti-IL-10 PE (Caltag Laboratoires), using the Fix & Perm reagent (Caltag Laboratoires) (Almeida *et al*, 1999; Bueno *et al*, 2001). Cytokine-directed mAbs were used at saturating concentrations and conditions. Samples stimulated under the same culture conditions in the absence of BFA showed undetectable cytokine levels. Cells labelled with isotype control mAbs were included to determine background fluorescence. The evaluation of cytokine production was based on the percentage of cytokine expressing cells. Cytokine production by T lymphocytes was also determined by flow cytometry, as percentages of cells expressing cytokines upon stimulation with PMA plus ionomycin (Sigma Chemical, St Louis, MO, USA) (Rostaing *et al*, 1999). To avoid ionophore-induced release of prostaglandins and thromboxane by platelets (Knapp *et al*, 1977) present in whole-blood samples, cultures of PBMCs were performed. Briefly, PBMCs were isolated from heparinised venous blood by density centrifugation on lympholyte (Cedarlane Labs, Hornby, Canada), as previously described (Della Bella *et al*, 2001). The cells were suspended at a concentration of 1 × 10^6^ cells ml^−1^ in complete culture medium consisting of RPMI 1640 supplemented with 10% heat-inactivated AB human serum, 2 mM glutamine, 50 U ml^−1^ penicillin, 50 *μ*g ml^−1^ streptomycin, in the presence of 10 *μ*g ml^−1^ BFA and in the presence or absence of 25 ng ml^−1^ PMA and 1 *μ*g ml^−1^ ionomycin. Cultures were incubated for 18 h at 37°C in a 5% CO_2_ humid atmosphere in a sterile environment. Doses of PMA and ionomycin and incubation time were optimised in preliminary experiments. After incubation, the cells were processed for flow cytometric analysis as described above. The percentage of T lymphocytes, identified based on the surface expression of CD3, expressing type 1 cytokines IL-2 and IFN-*γ*, and type 2 cytokine IL-4, was evaluated.

### Measurement of plasma levels of VEGF, IL-10 and spermine

Patient and control plasma samples were obtained by centrifugation of heparinised peripheral blood, and stored at –20°C. A commercial ELISA kit was used to measure plasmatic concentrations of VEGF (R&D Systems, Minneapolis, MN, USA), according to the manufacturer's instructions. The IL-10 ELISA was performed using IL-10-specific mAb 9D7 and biotinylated 12G8 (Endogen, Woburn, MA, USA). Plasmatic spermine concentrations were determined by reversed-phase high-performance liquid chromatography after derivatisation with dansyl chloride, as described (Marchesini *et al*, 1992).

### Effects of spermine on *in vitro* cultured DCs

Monocyte-derived DCs were generated from the adherent fraction of PBMCs obtained from healthy control subjects, as described (Bender *et al*, 1996). Briefly, PBMCs were allowed to adhere for 2 h in culture flasks, nonadherent cells were gently removed, and the remaining cells were cultured in RPMI 1640 containing 10% heat-inactivated FCS (Euroclone, Wetherby, West York, UK) and supplemented with 800 U ml^−1^ GM-CSF (Novartis, Italy) and 10 ng ml^−1^ IL-4 (PeproTech, London, UK). Cultures were fed every other day with fresh medium and cytokines. The effects of spermine on DC differentiation were examined by adding spermine (Sigma-Aldrich, St Louis, MO, USA) at the initiation of culture in parallel cultures. Aminoguanidine was added to cells along with spermine to inhibit the action of polyamine oxidase. Concentrations of spermine ranging from 0.5 to 4 *μ*M were used, because these levels were reported to have immunomodulatory effects (Haskò *et al*, 2000), and were relevant to plasmatic ranges occurring in our patients. Dendritic cells were collected at day 5. The immunophenotype of DCs was analysed by three-colour FACS analysis with 5000–10 000 events acquired for each sample. Endocytosis was assessed by the uptake of FITC-dextran (Sallusto *et al*, 1995). Dendritic cells were incubated for 1 h at 37°C in the presence of 1 mg ml^−1^ FITC-dextran (70 000 m.w.; Molecular Probes, Eugene, OR, USA). Control cells were incubated on ice for 1 h. After extensive washing, the cells were analysed by single-colour FACS analysis. Results were expressed as the difference between mean fluorescence intensity (MFI) obtained at 37 and 0°C (negative control). Phagocytosis of apoptotic cells by DCs was analysed by flow cytometry as reported by Bellone *et al* (1997) with some modifications. Briefly, allogeneic monocyte-depleted PBMCs were labelled with 0.5 *μ*M chloro-methyl-fluorescein-diacetate (CMFDA; Molecular Probes) for 30 min at 37°C, washed extensively and incubated for 24 h in medium supplemented with 10 *μ*M H_2_O_2_ to induce apoptosis. Chloro-methyl-fluorescein-diacetate-labelled apoptotic cells were then incubated with DCs at a ratio of 1 : 1 at 37°C. After 2 h, cells were washed and treated with 0.05% trypsine/0.02% EDTA for 3 min to disrupt cell – cell binding. Phagocytosis was quantified by flow cytometry as the percentage of CMFDA+ DCs. To evaluate their allostimulatory capacity, DCs were cocultured for 5 days with allogeneic monocyte-depleted PBMCs, at a ratio of 1 : 20. During the last 6 h of culture, 20 *μ*M 5-bromo-2′-deoxyuridine (BrdU; Sigma Chemical Company, St Louis, MO, USA) was added, and the proliferation of allogeneic T lymphocytes was evaluated by flow cytometry as BrdU incorporation by CD3+ cells, as described by Toba *et al*. Data are expressed as the percentage of proliferating BrdU+ T lymphocytes. IL-12 production was assessed by stimulating monocyte-derived DCs for 24 h with soluble recombinant CD40L (1 *μ*g ml^−1^; Alexis Biochemicals, San Diego, CA, USA). The IL-12 p70 ELISA was performed using p70-specific mAb 20C2 and biotinylated C8.6 (Endogen).

### Statistical analysis

Comparisons of samples to establish the statistical significance of difference were determined by the two-tailed Student's *t*-test for independent samples. The paired *t*-test and the linear regression analysis were also used when indicated. Results were considered to be statistically significant when *P* was ⩽0.05.

## RESULTS

### Quantitation of PB DCs and DC precursors

**Figure 1:**
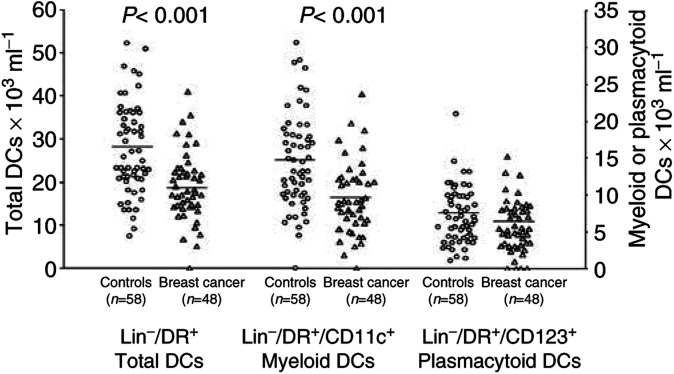
Absolute number of PB DCs in breast cancer patients compared with controls. A significant decrease in PB Lin-/HLA-DR+ cells (left axis) and myeloid DCs (right axis) was observed in cancer patients. Whole blood was stained with a cocktail of FITC-conjugated mAbs recognising CD3, CD14, CD16, CD19 and CD20, and with PerCP-conjugated anti-HLA-DR mAb; myeloid DCs were identified as lineage-/HLA-DR+/CD11c+ cells, plasmacytoid DCs (right axis) as lineage-/HLA-DR+/CD123+ cells. Absolute DC counts were then determined indirectly by multiplying the percentage of DCs in the mononuclear gate times the sum of the lymphocyte and monocyte determined on a differential blood cell counter. Each symbol represents a single sample. Open circles: control subjects; open triangles: breast cancer patients. Mean values represented by horizontal lines in each series. *P*-values were determined using the *t*-test for independent samples, patients compared with controls.

The percentage of DCs, identified as Lin-/HLA-DR+ cells, in the mononuclear cell population was significantly lower in the peripheral blood of breast cancer patients compared with healthy controls (breast cancer: 0.87±0.04% (mean±s.e.m.) of the mononuclear cells *vs* controls: 1.45±0.06%; *P*<0.001). The number of DCs in the blood, estimated by using the percentages generated by FACS analysis and the absolute mononuclear cell count, was also significantly lower in cancer patients than in controls (19.37±1.10 × 10^3^ ml^−1^ *vs* 27.71±1.59, *P*<0.001) (Figure 1

). Within the Lin-/HLA-DR+ cells, the percentage of CD11c+ DCs tended to be lower in cancer patients (49.7±2.1 *vs* 54.6±2.2%, *P*=n.s.) and that of CD123+ DCs tended to be higher (32.1±1.8 *vs* 27.5±1.8%, *P*=n.s.). This resulted in a significant decrease of PB myeloid Lin-/HLA-DR+/CD11c+ DCs in breast cancer patients compared with control subjects (9.61±0.68 × 10^3^ ml^−1^ *vs* 14.80±0.95, *P*<0.001), while the number of PB plasmacytoid Lin-/HLA-DR+/CD123+ DCs was unaffected. There was no correlation between DC number and tumour size or stage in patients with invasive breast cancer. The decrease in the number of DCs in the blood was already apparent, although not significant, in the few patients with carcinoma *in situ* included in the study (20.17±2.92 × 10^3^ ml^−1^), while DC number was unaffected in the patients with benign breast tumours (26.66 ± 5.37 × 10^3^ ml^−1^). The frequency of CD34+ cells in the blood of cancer patients was significantly higher than in control subjects (1.13±0.32%, *n*=7 *vs* 0.57±0.06, *n*=12; *P*=0.041). Finally, the frequency of CD14+ monocyte precursors did not differ between patients and controls (5.89±2.02, *n*=38, and 6.06±0.02, *n*=14, respectively).

### Immunophenotype of PB DCs

**Figure 2:**
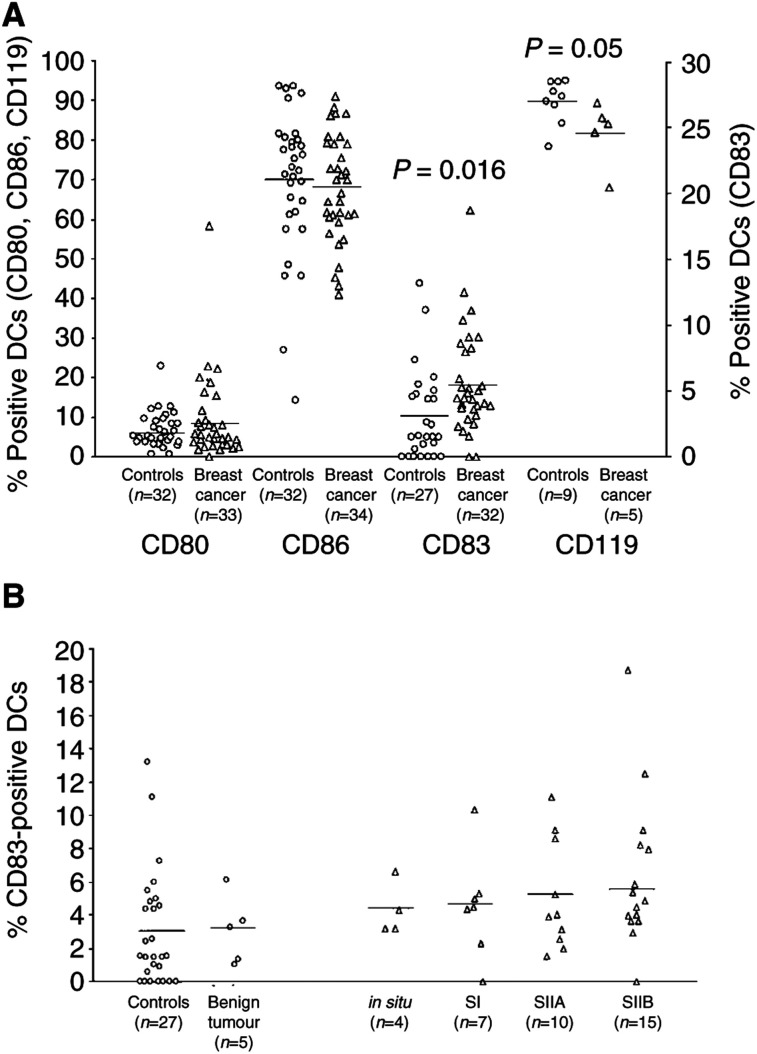
Immunophenotype of PB DCs. (**A**) The activation state of Lin-/HLA-DR+ DCs, assessed as the expression of the costimulatory molecules CD80 and CD86 (*left axis*), was similar in patients and controls, while the frequency of mature DCs was significantly higher in breast cancer individuals, as assessed by higher percentages of Lin-/HLA-DR+ cells expressing CD83 (right axis) and lower percentages of Lin-/HLA-DR+ cells expressing CD119 (left axis). (**B**) The percentage of mature DCs, identified as Lin-/HLA-DR+ cells expressing the CD83 maturation marker, increased progressively with the severity of breast cancer. Each symbol represents a single sample. Open circles: control subjects; open triangles: breast cancer patients. Mean values are represented by horizontal lines in each series. *P*-values were determined using the *t*-test for independent samples, patients compared with controls.

The activation state of PB DCs, assessed as the expression of the costimulatory molecules CD80 and CD86 on Lin-/HLA-DR+ cells, did not significantly differ between patients and controls, although cancer patients presented a slightly higher percentage of CD80+ DCs (9.15±1.88% of the DCs *vs* 6.69±0.94%) (Figure 2A

). Otherwise, analysis of the maturation state of Lin-/HLA-DR+ DCs, evaluated as the expression of CD83 molecule, showed that breast cancer patients had a significantly higher percentage of mature CD83+ DCs compared with control subjects (5.23±0.57 *vs* 3.00±0.75%; *P*=0.019). As shown in Figure 2B, DC maturation appeared correlated to the clinical stage of disease, since the percentage of CD83+ DCs tended to increase progressively with the severity of breast cancer (carcinoma *in situ*: 4.31 ± 0.81; stage I: 4.91 ± 2.00; stage IIA: 5.11 ± 1.05; stage IIB: 5.46 ± 0.83). The percentage of CD83+ DCs in patients with benign tumours of the breast was absolutely unaffected (3.07 ± 0.92) as compared with controls. To evaluate the early-maturation stage of DCs, in a few cases we also analysed DC surface expression of the IFN-*γ* receptor (CD119), which is progressively down-regulated on maturing DCs (Kalinski *et al*, 1999). Breast cancer patients indeed had a significant lower percentage of CD119+ Lin-/HLA-DR+ DCs compared with healthy controls (82.00 ± 3.63 of the DCs *vs* 89.92 ± 1.85%; *P*=0.05).

### Effects of LPS stimulation on activation and maturation of DCs

To assess the ability of PB DCs from breast cancer patients to be activated following stimulation, we exposed whole-blood samples to LPS for 5 h, and subsequently analysed the expression of costimulatory molecules and CD83 on DCs. In both patients and controls, this short-period incubation with LPS induced a statistically significant increase in the percentage of both CD80+ Lin-/HLA-DR+ DCs and CD83+ Lin-/HLA-DR+ DCs (patients and controls: paired *t*-test *P*<0.001). LPS also induced an overexpression of CD86 above the constitutive levels in both the groups, measured as MFI (patients and controls: paired *t*-test *P*<0.001). The activation and maturation response of DCs to LPS did not differ between patients and controls, with similar percentages of CD80+ DCs (patients: 48.43 ± 4.78% of DCs *vs* controls: 41.17 ± 2.90%) and CD83+ DCs (75.94 ± 3.17 *vs* 71.21 ± 2.61%), and a similar DC expression of CD86 (MFI: 398.36 ± 39.15 *vs* 349.67 ± 27.45%) observed in both the groups in LPS-exposed blood samples. Incubation of whole-blood samples in plastic tubes in the presence of culture medium without LPS induced a slight activation and maturation of DCs, which emphasised differences between patients and controls, resulting in significantly higher percentages of CD80+ DCs (9.80 ± 1.72 *vs* 3.86 ± 0.77%, *P*=0.004) and CD83+ DCs (14.86 ± 3.51 *vs* 4.53 ± 1.42%, *P*=0.011), and higher DC expression of CD86 (163.14 ± 20.33 *vs* 86.47 ± 10.11%, *P*=0.028) in breast cancer patients than in control subjects.

### Cytoplasmic cytokine expression in PB DCs

**Figure 3:**
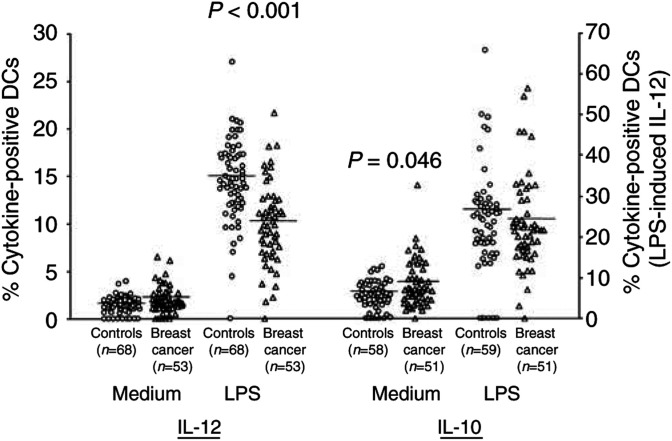
Cytokine production by peripheral blood DCs in breast cancer patients compared with control subjects. A significantly lower percentage of PB DCs producing IL-12 upon LPS stimulation was observed in cancer patients. After incubation for 5 h in the presence or absence of LPS with BFA added during the last 4 h, whole-blood samples were stained with a cocktail of FITC-conjugated mAbs recognising CD3, CD14, CD16, CD19 and CD20, and with PerCP-conjugated anti-HLA-DR mAb. Intracellular accumulation of cytokines within DCs, identified as lineage-/HLA-DR+ cells, was evaluated after staining with PE-conjugated mAbs directed against either IL-12 or IL-10. Each symbol represents a single sample. Open circles: control subjects; open triangles: breast cancer patients. LPS-induced IL-12 values referred to the right axis; all the other values to the left axis. Mean values represented by horizontal lines in each series. *P*-values were determined using the *t*-test for independent samples, patients compared with controls.

**Figure 4:**
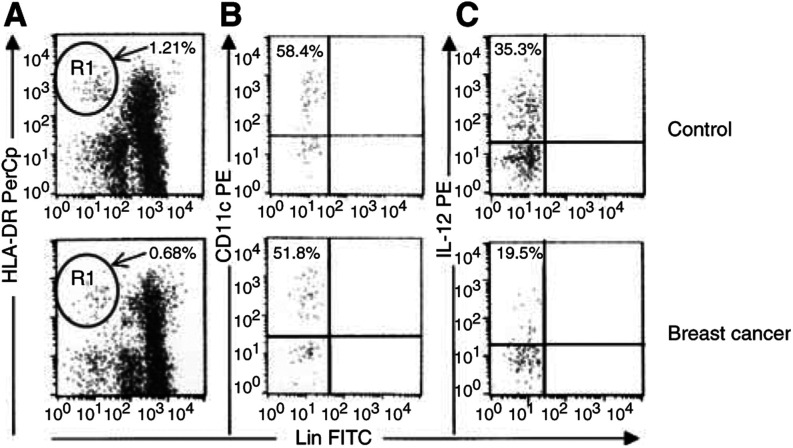
Identification and characterisation of PB DCs in whole peripheral blood samples. Comparison between representative flow cytometric analyses from a control subject (upper line) and a breast cancer patient (lower line). (**A**) Gated on a mononuclear cell analysis region, DCs were identified on the basis of their lack of labelling for the lineage markers CD3, CD14, CD16, CD19 and CD20, but positive staining for HLA-DR (R1). (**B**) Gated on R1 events, myeloid DCs were identified for their surface expression of CD11c. (**C**) Gated on R1 events, PB DCs producing IL-12 upon LPS stimulation were identified for their intracellular accumulation of the cytokine.

Owing to the central role played by regulatory cytokines produced by DCs in directing immune responses, we further asked whether the ability of PB DCs from breast cancer patients to produce cytokines was impaired. We incubated whole-blood samples with or without LPS in the presence of BFA for 5 h, and subsequently analysed the expression of intracellular cytokines in unseparated Lin-/HLA-DR+ DCs. In both the groups, incubation of peripheral blood samples with LPS induced a significant increase in the percentage of DCs expressing either IL-12 or IL-10 (patients and controls: paired *t*-test *P*<0.001). As shown in Figure 3

, while the unstimulated expression of IL-12 was similar in the two groups, the percentage of DCs expressing IL-12 upon LPS stimulation was significantly lower in breast cancer patients than in healthy controls (23.75 ± 1.35 *vs* 34.28 ± 1.38%, *P*<0.001). A representative flow cytometric analysis is presented in Figure 4

. The reduction in IL-12 expression in cancer patients was also evident when we compared the mean fluorescence intensity for IL-12 staining in patients and controls (60.9 ± 5.6 *vs* 86.4 ± 11.2%, *P*=0.036). The percentage of DCs expressing cytoplasmic IL-10 was significantly higher in cancer patients in unstimulated conditions (3.70 ± 0.35 *vs* 2.65 ± 0.22%, *P*=0.046), while it was similar to control subjects upon LPS stimulation. As for the DC count, the decrease of DCs expressing IL-12 following LPS stimulation was not observed in patients with benign tumour of the breast (39.24 ± 10.12), and was only slight in patients with carcinoma *in situ* (32.28 ± 3.39). A significant positive correlation was observed between the percentage of DCs expressing IL-12 and the percentage of DCs expressing CD119 on their surface (*r*=0.543, *n*=13, *P*=0.05). No significant correlation was found between cytokine expression by DCs and size or stage of the tumour, or between the percentages of DCs expressing IL-12 and IL-10.

### Cytoplasmic cytokine expression in T lymphocytes

**Figure 5:**
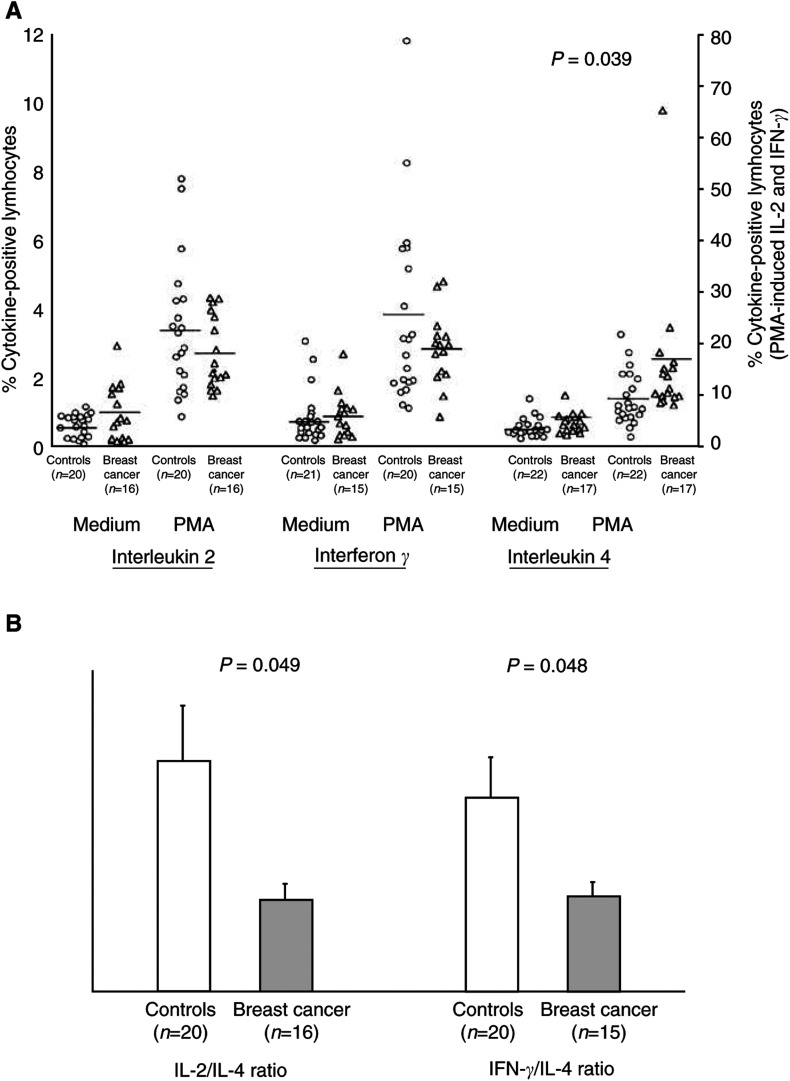
Cytokine production by T lymphocytes in breast cancer patients compared with control subjects. PBMCs were cultured in the presence of BFA with or without PMA plus ionomycin for 18 h. At the end of the cultures, cells were stained with FITC-conjugated anti-CD3 mAb, and intracellular accumulation of cytokines within CD3+ T lymphocytes was evaluated after staining with PE-conjugated mAbs directed against either IL-2 or IFN-*γ* or IL-4. (**A**) Each symbol represents a single sample. Open circles: control subjects; open triangles: breast cancer patients. Values of PMA-induced IL-2 and IFN-*γ* referred to the right axis, all the others to the left axis. Mean values are represented by horizontal lines in each series. *P*-values were determined using the *t*-test for independent samples, patients compared with controls. (**B**) Profile of cytokine production by T lymphocytes, expressed as type 1 to type 2 cytokine ratios. Both IL-2 to IL-4 and IFN-*γ* to IL-4 ratios were significantly reduced in breast cancer patients compared with controls. Open bars: control subjects; dark bars: breast cancer patients. *P*-values were determined using the *t*-test for independent samples, patients compared with controls.

To assess whether the ability of T lymphocytes from breast cancer patients to produce cytokines was also affected, we stimulated PBMCs with PMA and ionomycin in the presence of BFA, and analysed the intracellular expression of type 1 and type 2 cytokines by CD3+ lymphocytes. In both the groups, incubation of PBMCs with PMA and ionomycin induced a significant increase in the percentage of T lymphocytes expressing either IL-4 (patients: paired *t*-test *P*=0.003; controls: *P*<0.001) or IL-2 (patients and controls: *P*<0.001) or IFN-*γ* (patients and controls: *P*<0.001). As shown in Figure 5A

, the percentages of T lymphocytes expressing cytokines in unstimulated cultures were low and similar in patients and controls. Upon PMA stimulation, the percentage of T lymphocytes expressing IL-4 was significantly higher in breast cancer patients than in controls (2.36 ± 0.49 *vs* 1.37 ± 0.16%, *P*=0.039), while the percentages of CD3+ cells expressing either IL-2 or IFN-*γ* were slightly decreased in cancer patients. Since the balance between type 1 and type 2 cytokines is thought to be more relevant than absolute values of single cytokines in driving immune responses, the ratios between cytokine-expressing lymphocytes were also considered. As shown in Figure 5B, both the ratio between IL-2+ and IL-4+ lymphocytes and the ratio between IFN-*γ* + and IL-4+ lymphocytes, in PMA-stimulated cultures, were significantly lower in breast cancer than in control subjects (IL-2/IL-4: 10.06 ± 1.61 *vs* 25.01 ± 5.97%, *P*=0.049; IFN-*γ*/IL-4: 10.43 ± 1.47 *vs* 21.07 ± 4.18%, *P*=0.048). No correlation was observed between cytokine expression by T lymphocytes and DC number or cytokine expression by DCs.

### Effects of surgical removal of the tumour on PB DCs

**Figure 6:**
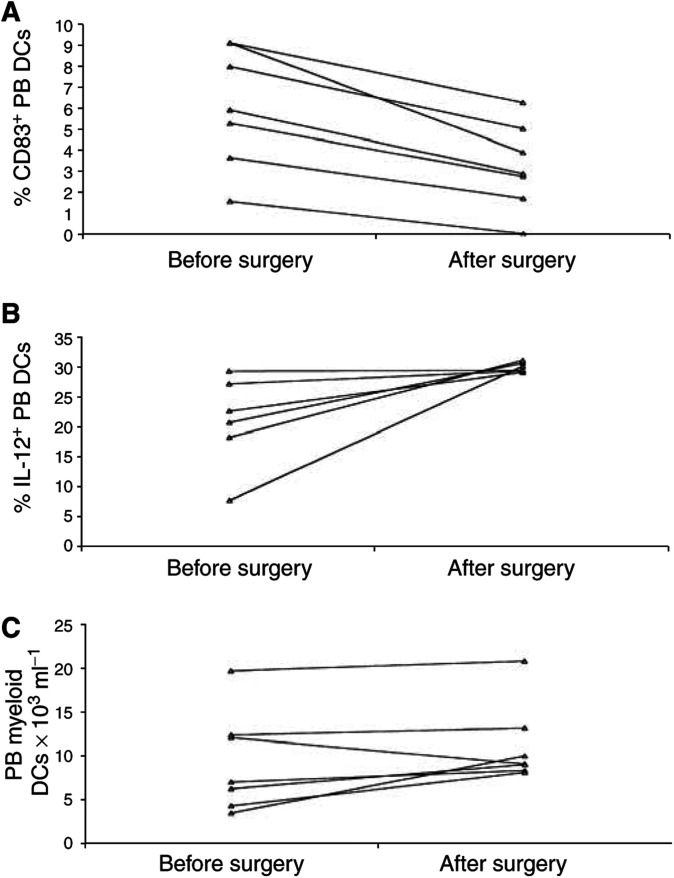
Effects of surgical removal of the tumour on PB DCs. At 4 weeks after surgery, (**A**) the percentage of CD83+ PB DCs significantly decreased (*P*=0.001), with a reduction of mature PB DCs observed in all the patients; (**B**) the percentage of PB DCs expressing IL-12 upon LPS stimulation significantly increased (*P*=0.042), with a complete normalisation observed in all the patients; (**C**) the number of myeloid DCs in the peripheral blood of the patients only slightly increased (*P*=n.s.). Methods as described in Figures 2–4 Each symbol represents a single sample. *P*-values were determined using the *t*-test for paired samples.

To assess the impact of surgical resection of the tumour, we compared PB DCs in seven breast cancer patients before and 4 weeks after the surgery, but prior to adjuvant chemotherapy or radiation therapy. As shown in Figure 6

, removal of the tumour resulted in normalisation of both the percentage of CD83+ DCs (pre: 6.06 ± 1.08%; post: 3.21 ± 0.78%; paired *t*-test: *P*=0.001) and the percentage of DCs expressing IL-12 upon LPS stimulation (pre: 21.18 ± 3.11%; post: 30.07 ± 0.33%; *P*=0.042). Removal of the tumour also induced a decrease, although not significant, of the percentage of CD80+DCs (pre: 10.52 ± 3.53%; post: 6.41 ± 1.70%; *P*=n.s.). At the time of blood collection, only a partial non-significant increase of both the proportion of DCs (pre: 0.72 ± 0.09% of the mononuclear cell; post: 0.77 ± 0.05%) and the absolute number of myeloid DCs (pre: 9.24 ± 2.17 × 10^3^/ml; post: 11.13 ± 1.72) was observed.

### Correlation between PB DC functions and plasmatic levels of tumour-derived mediators

To evaluate whether alterations in PB DCs could depend on tumour-related soluble factors, we analysed the levels of VEGF, IL-10 and spermine in the plasma of nine patients compared with an equal number of control subjects. The plasmatic levels of VEGF (breast cancer: range 31.2 – 177.9 pg ml^−1^; controls: 43.7 – 59.6), IL-10 (breast cancer: 20.9 – 31.1; controls: 16.9 – 21.7) and spermine (breast cancer: 0.25 – 4.05; controls: 0.66 – 2.51) did not significantly differ between groups. However, a significant negative correlation was found between the plasmatic concentration of spermine and the percentage of DCs expressing IL-12 upon stimulation (*r*= −0.768, *n*=9, *P*=0.016). Furthermore, the levels of spermine tended to be directly correlated with the percentage of CD83+ DCs, although this correlation did not reach statistical significance. No correlation was observed between the plasmatic concentration of VEGF or IL-10 and any DC parameter.

### Effects of spermine on cultured DCs

**Figure 7:**
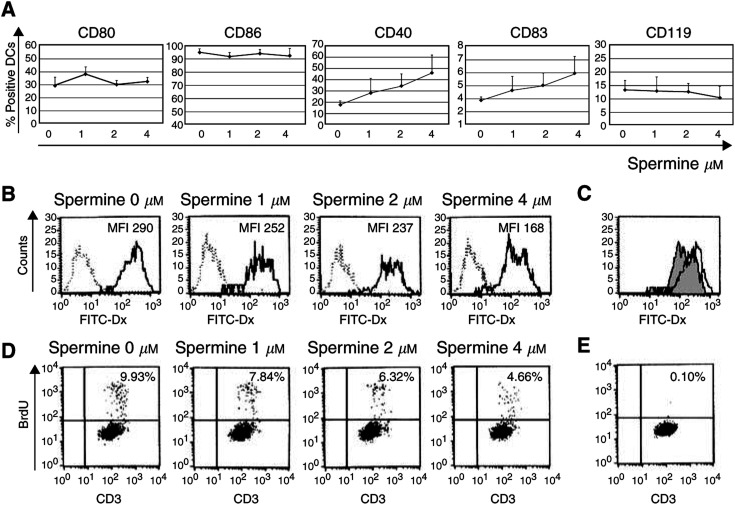
Effects of spermine on *in vitro* cultured DCs. Monocyte-derived DCs were generated from the adherent fraction of PBMCs obtained from healthy control subjects in the presence of IL-4 and GM-CSF for 5 days. Spermine at the indicated concentrations was added at the initiation of culture and maintained throughout. (**A**) Exposure of maturing DCs to spermine affected their immunophenotype, promoting a dose-dependent mild increase of DC expression of CD83 and CD40. Data shown are mean ± s.e.m of four independent experiments. (**B**) The addition of spermine to maturing DCs impaired their mannose-receptor-mediated FITC-dextran endocytosis, as assessed by a dose-dependent mean fluorescence intensity reduction. Dashed lines indicate FITC-dextran endocytosis at 0°C (negative control), and solid lines represent FITC-dextran endocytosis at 37°C at the indicated spermine concentrations. This result is representative of four independent experiments. (**C**) Same experiment as (**B**). Comparison between FITC-dextran endocytosis by DCs cultured in the absence (white histogram) or presence (grey histogram) of 4 *μ*M spermine. (**D**) Exposure of maturing DCs to spermine affected their allostimulatory capacity. Lymphocyte proliferation was measured by flow cytometry as BrdU incorporation by CD3+ cells. The percentages of proliferating allogenic T lymphocytes after coculture for 5 days with DCs generated in the presence of the indicated spermine concentrations are presented. This result is representative of four independent experiments. (**E**) BrdU incorporation by T lymphocytes cultured for 5 days in the absence of DCs (negative control) is shown.

To evaluate whether spermine could induce alterations in DC differentiation and function, we cultured monocyte-derived DCs from healthy donors in the presence or absence of spermine. This polyamine did not affect the efficiency of generation of DCs from monocytes, since similar numbers of DCs were obtained in its presence or absence (data not shown). Preliminary results obtained from four independent experiments seem to indicate that exposure of maturing DCs to spermine slightly promotes their maturation, as assessed by a dose-dependent mild increase of DC expression of CD83 – at levels similar to those observed *in vivo* – and of CD40. The results of immunophenotype analysis are reported in Figure 7

**Figure 8:**
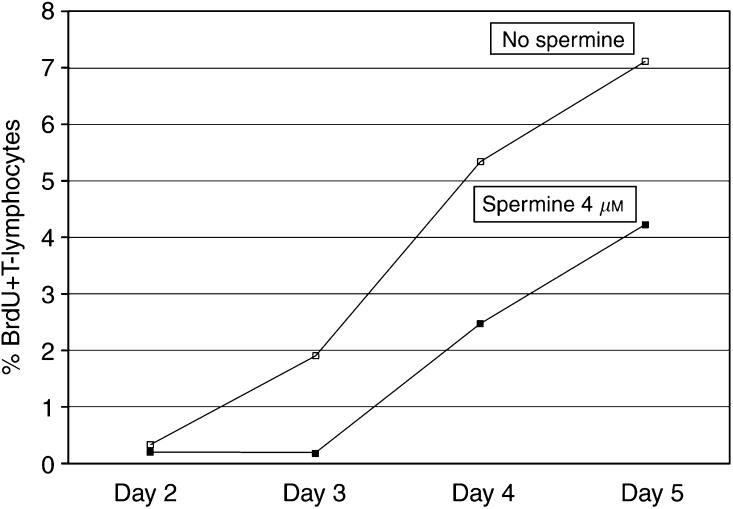
Analysis of T-cell division induced at different days by allogenic unconditioned (white squares) or spermine-conditioned (black squares) DCs. The decrease in T-cell stimulatory function seen for DCs cultured with spermine was not due to a different cell kinetics. Methods as in Figure 7; results expressed as percentages of proliferating allogenic T lymphocytes.

. As illustrated in the same figure, DCs generated in the presence of spermine showed a reduced capacity to capture antigens, as assessed by a reduced mannose-receptor-mediated endocytosis (MFI FITC-dextran, mean±s.e.m. from 428.9 ± 81.9 to 315.7 ± 86.5%). The reduced capacity of spermine-conditioned DCs to capture antigens was also measurable as reduced phagocytosis of apoptotic cells (% CMFDA+ DCs: from 18.3 ± 2.1 to 11.7 ± 2.9%). Moreover, DCs exposed to spermine exibited a dose-dependent impairment of their allostimulatory capacity, as assessed by reduction in the percentage of BrdU+ proliferating allogenic T lymphocytes on day 5 of culture (% BrdU+ CD3+ cells: from 11.4 ± 2.59 to 7.7 ± 2.1%). A representative experiment is reported in Figure 7. It was possible that the decrease in T-cell stimulatory function seen for DCs cultured with spermine reflected the fact that the T-cell division in these cultures already had peaked, since spermine-conditioned DCs were more mature and may have activated the T cells more rapidly. This possibility was excluded by a kinetic study, shown in Figure 8

. Finally, exposure of maturing DCs to spermine partially inhibited the ability of these cells to produce IL-12 upon stimulation, as detected by ELISA measurement of the cytokine in culture supernatants (from 328 ± 124 to 181 ± 83 pg ml^−1^).

## DISCUSSION

In the present study, we evaluated PB DCs in patients with breast cancer compared with control subjects. We applied flow cytometric methods that allow the enumeration and the immunophenotypical as well as functional characterisation of DCs directly in whole peripheral blood samples (Robinson *et al*, 1999; Arpinati *et al*, 2000; Upham *et al*, 2000). With this method, we observed that Lin-/HLA-DR+ DCs are significantly reduced in the peripheral blood of breast cancer patients compared with healthy donors. The reduction was already apparent, although not significant, in patients with *in situ* breast carcinoma, and was more severe and statistically significant in patients with invasive carcinoma. Within the group of invasive breast cancer, which was composed of patients in the early stage of the disease (I, IIA and IIB), we did not find a correlation between DC count and stage of cancer. The close correlation between DC reduction and cancer was emphasised by the finding that the number of PB DCs was unaffected in the few women who, following surgical removal, were diagnosed as affected by benign breast tumour. A similar reduction of Lin-/HLA-DR+ DCs had been observed by Gabrilovich *et al* in the peripheral blood of patients with several malignant neoplasias, including a group of subjects with breast cancer at various stages, from I to IV (Almand *et al*, 2000). Our study confirms and extends these previous observations, focusing on a larger group of early-stage breast cancer patients. Furthermore, Gabrilovich *et al* estimated blood DC number by extrapolating from the yield of DCs obtained after multistep purification, while we performed DC count directly on whole blood, without any *ex vivo* enrichment. The results obtained with this procedure, that more directly reflects the *in vivo* situation, may corroborate the possible role of DC alterations in the clinical evolution of malignancies. Together with the reduction of Lin-/HLA-DR+ DCs, we observed a statistically significant increase of CD34+ cells in the peripheral blood of patients with invasive breast cancer. This increase could be related to tumour-derived factors that may either promote the mobilisation of CD34+ cells from the bone marrow, such as GM-CSF (Garrity *et al*, 1997), or inhibit DC maturation from CD34+ precursors, such as VEGF, M-CSF and IL-6 (Almand *et al*, 2000). In patients with head and neck squamous cell carcinoma, an increased number of CD34+ progenitor cells endowed with natural suppressor activity is present in the peripheral blood and within the tumour tissue (Garrity *et al*, 1997; Lathers *et al*, 1999). To further investigate the significance of the increase of PB CD34+ cells in breast cancer patients, ongoing studies are planned to characterise the immunophenotype and the functional activity of these cells in our patients.

Since human PB DCs can be divided, on the basis of expression of the *β*-integrin CD11c (Robinson *et al*, 1999), into two subpopulations that differ in the origin, expression of phenotypic markers, route of activation and immunological activity, we evaluated their distribution in our patients. We found that the number of myeloid DCs, and not plasmacytoid DCs, was significantly decreased in breast cancer patients compared to controls. This alteration may have a relevant impact on immune defences against cancer. In fact, CD11c+ myeloid DCs originate from myeloid bone marrow precursors, express myeloid antigens, and upon stimulation produce high amounts of IL-12, which in turn promotes cell-mediated immune responses that are crucial for the detection and elimination of malignant cells (Cella *et al*, 1996; Rissoan *et al*, 1999; Robinson *et al*, 1999). CD11c- plasmacytoid DCs originate from lymphoid bone marrow precursors, lack myeloid markers, express high levels of the IL-3-receptor-*α* chain (CD123) and secrete lower amounts of IL-12 (Rissoan *et al*, 1999; Robinson *et al*, 1999; Reid *et al*, 2000; Liu *et al*, 2001). Although it is now believed that each DC subset has a certain degree of flexibility in directing T-cell responses (Liu *et al*, 2001; Shortman and Liu, 2002), myeloid DCs still remain higher producers of IL-12 than plasmacytoid DCs and key elements in the induction of cell-mediated immune responses.

Since the presence of costimulatory molecules on DC surface is crucial in determining whether engaged T lymphocytes will become anergic or develop productive immunity (Schultz *et al*, 1996), we evaluated the expression of CD80 and CD86 on Lin-/HLA-DR+ DCs in our patients, but we did not find significant differences compared with healthy controls. Since another important aspect of DC function is the maturation state, we also evaluated the expression of the CD83 molecule, a widely used DC maturation marker supposed to be involved in antigen presentation and cellular interactions (Zhou and Tedder, 1995; Lechmann *et al*, 2002). We observed that the percentage of mature CD83+ DCs is higher in patients with invasive breast cancer compared with subjects of all other groups (*in situ* carcinoma, benign breast neoplasms and healthy controls). Interestingly, CD83+ DCs have been described in the peritumoral areas of breast cancer samples, while absent in the normal breast tissue (Bell *et al*, 1999). We are planning further studies to investigate the relationships existing between DCs in peripheral blood and in tumour tissue in our patients. In normal conditions, PB CD83+ DCs are infrequent because DC maturation and CD83 expression follow antigen capture and exposure of DCs to activating stimuli in peripheral tissues. The high percentage of Lin-/HLA-DR+ DCs expressing CD83 observed in breast cancer patients could be induced by tumour-derived factors, as suggested by the observation that the increased expression of CD83 by PB DCs was completely reverted in our patients 4 weeks after surgical removal of the tumour. Moreover, *in vitro* tumour supernatants are able to promote rapid maturation of DCs with upregulation of CD83 molecule expression (Kiertscher *et al*, 2000). Finally, factors present in the tumour environment *in vivo*, such as polyamines or prostaglandins, might also interfere with the maturation and/or function of DCs (Haskò *et al*, 2000; Harizi *et al*, 2002). In fact, we found that exposure of *in vitro* maturing DCs to spermine enhanced the expression of CD83, suggesting that this tumour-derived polyamine could be involved in the phenotypic maturation of PB DCs observed *in vivo*. Functional impairment of DCs have also been described in haematologic diseases, in particular in chronic and acute myeloid leukaemia, in which defects in antigen processing (Dong *et al*, 2003) and in allostimulatory activity (Mohty *et al*, 2001) have been related to the expression by DCs of the same cytogenetic abnormalities expressed by freshly isolated leukaemic cells. It has been suggested that the defects in antigen processing observed in chronic myeloid leukaemia DCs may be related to the underlying cytoskeletal changes induced by the expression of the BCR-ABL fusion gene (Dong *et al*, 2003). At present, we cannot exclude the fact that cytoskeletal alterations may similarly contribute to impaired function of DCs in breast cancer patients.

Owing to the important role of regulatory cytokines of DC origin in the induction and activation of immune responses, we evaluated the cytofluorimetric DC expression of IL-12 and IL-10, both in basal and stimulated conditions. We used LPS as the DC stimulator because LPS, with or without IFN-*γ*, has been reported to be the optimal stimulus for analysis of cytokine expression by DCs in whole-blood samples (Almeida *et al*, 1999; Bueno *et al*, 2001), and because TLR4, which is required for the response to LPS, is selectively expressed on myeloid CD11c+ DCs (Krug *et al*, 2001). We found that DC constitutive production of IL-10 was increased in patients with invasive cancer, while that of IL-12 was similar to controls. On the contrary, when we evaluated LPS-induced expression of cytokines, we observed an impaired expression of IL-12 by PB DCs from breast cancer patients. The possibility that the reduction in IL-12-producing cells simply reflects the reduction in myeloid DCs seems unlikely because the reduction in the percentage of IL-12-positive DCs largely overcomes the reduction in the percentage of CD11c+ myeloid DCs. Moreover, because the reduction of IL-12 expression in our patients is reversed by tumour excision, we hypothesised that this defect could be directly dependent on cancer mass and on its environmental factors, such as polyamines. This possibility is supported by the inverse correlation existing between plasmatic levels of spermine and the percentage of PB DCs expressing IL-12 in our patients. The ability of spermine to interfere with IL-12 production seems to be confirmed by our preliminary experiments on DCs cultured *in vitro* in the presence of spermine at concentrations readily achieved *in vivo*. The impairment of IL-12 production could also be correlated with the increased maturation state of cancer DCs. In fact, it is known that mature CD83+ DCs have a reduced IL-12-producing capacity, resulting mainly from their impaired responsiveness to IFN-*γ*, which is a cofactor in IL-12 induction (Kalinski *et al*, 1999). Accordingly, in our study, we found that the percentage of Lin-/HLA-DR+ DCs expressing IFN-*γ* receptor (CD119) was significantly reduced in cancer patients. Furthermore, the percentage of DCs expressing IL-12 was directly correlated with the percentage of DCs expressing CD119 on their surface. The reduced capacity of PB DCs to express IL-12 upon LPS *in vitro* stimulation in our breast cancer patients was specific for this cytokine, and could not be ascribed to a general hyporesponsiveness of these cells to LPS. In fact, LPS-induced upregulation of CD80, CD86 and CD83, and the expression of IL-10 were similar in cancer patients and healthy controls.

A perturbation of the cytokine network characterised by low levels of IL-12 and normal or high levels of IL-10 has been previously reported in patients with neoplasms of various organs (O'Hara *et al*, 1998; Jacobs *et al*, 1998; Merendino *et al*, 1999); it has been suggested that this imbalance in IL-12 and IL-10 levels could explain the shift from type 1 to type 2 cytokine production by T lymphocytes known to occur in cancer patients (Elässer-Beile *et al*, 1993; Clerici *et al*, 1994; Clerici *et al*, 1997, 1998). Actually, we found that T-helper lymphocytes from our cancer patients were polarised towards the Th2 phenotype, suggesting that altered production of regulatory cytokines by DCs may affect adaptive immune responses.

In summary, our results seem to suggest that breast cancer affects DC maturation following two pathways. The first one could affect early stages of DC differentiation and could be responsible for the increased frequency of CD34+ cells and decreased number of Lin-/HLA-DR+ DCs observed in the peripheral blood of breast cancer patients (Almand *et al*, 2000). The second pathway could affect later stages of DC differentiation, and could be responsible for the shift to a mature-like phenotype and for the impaired IL-12 production by PB DCs. We suggest that these two mechanisms could concomitantly act in breast cancer to prevent the establishment of an effective antitumour immune response.
